# Aiding Clinical Decision-Making at the Individual and Community Level Using Mobile Sensor Data: Study Protocol for an Experimental Design

**DOI:** 10.7759/cureus.71398

**Published:** 2024-10-13

**Authors:** Vikas Arora, Shashank S Mishra, Ashish Joshi, Manmohan Singhal, Jagannath Sahoo

**Affiliations:** 1 School of Pharmaceutical and Population Health Informatics, Faculty of Pharmacy, DIT University, Dehradun, IND; 2 School of Public Health, City University of New York, New York, USA; 3 Department of Pharmacology, Shobhaben Pratapbhai Patel School of Pharmacy and Technology Management, Narsee Monjee Institute of Management Studies, Mumbai, IND

**Keywords:** mhealth, mobile sensor data, physical activity, remote monitoring, sedentary lifestyle, sensor data

## Abstract

Background

Mobile sensor data can provide vital information about patients’ behavior and physical activity. In particular, the mobile accelerometer sensor data, if monitored effectively, can be used further for keeping watch on the physical activity of an individual and planning an intervention to improve it. These sensor data can also be used for improving clinical decision-making in sedentary lifestyle-related diseases. However, despite the availability of smartphone devices to the majority of the population, the sensor statistics captured by those devices are underused because of the lack of awareness about sensor metadata and the absence of clear-cut guidelines for using them.

Objective

The objective of this study is to study and encourage the use of the accelerometer sensor metadata of the mobile device in facilitating the delivery of interventions to enhance physical activity. At the end of the study, it should be able to lay down the basic guidelines for using the accelerometer sensor data of the mobile device for monitoring patients’ activity and aiding the clinical decision monitoring.

Methods

A sequential explanatory mixed-method research design will be used between July 2023 and December 2024. Firstly, individuals between the ages of 30 and 45 visiting the clinic of healthcare workers and having lifestyle-related diseases will be screened for a sedentary lifestyle with the pre-tested sedentary lifestyle measuring tool. After enrolling patients with a sedentary lifestyle in the study, physical activity-related accelerometer sensor data will be collected from them. Based on the initial data recorded, an individualized activity plan will be created. Periodic monitoring of physical activity levels will be conducted over a three-month period. Participants will receive a message from clinicians based on their current physical activity levels to encourage them to adhere to the prescribed regimen suggested by clinicians. The improvement in physical activity over the period will be monitored, and feedback from clinicians to improve the mechanism will be recorded.

Results

The proposed research will help in exploring the role of remote monitoring of sensor data in improving adherence to physical activity plans as prescribed by their clinicians. It will also help in studying the variables that influence the monitoring of remote data collection.

Conclusions

This study will help create a protocol to bring improvement in the physical activity levels of patients with a sedentary lifestyle and with confirmed hypertension and type 2 diabetes through remote capturing and monitoring of mobile sensor data, ongoing messaging, and facilitation through clinical decision-making.

## Introduction

Background and rationale 

Monitoring the physical activity of an individual has remained a challenging task for clinicians despite the availability of many self-reporting questionnaire assessment tools, primarily because of the lack of standardization in quantifying physical activities and recall bias [1]. However, in the last decade, with the introduction of smartphones and various electronic gadgets, measuring and quantifying physical activity has become a lot easier and more accurate. A study has established that a simple mobile phone intervention that uses accelerometer data can help reduce sedentary time and increase physical activity [2].

By the end of 2023, it is estimated that there will be around 500 million mobile users in India [3]. These mobile phones can be the potential sources of passive data collection about users without intruding on the personal space of the subjects in a timely manner [4]. Because of these inherent capabilities, mobile phones are being seen as a potential tool that can transform healthcare and reduce the burden on it by collecting continuous information about the patient and suggesting behavioral changes [5]. In continuation, there are pieces of evidence that suggest that mobile-based sensor data could be used effectively for predicting the causal interference and changes in the health status of the individual [6]. With the help of smartphones, a variety of personal data can be acquired easily and be used further for assessing the physical, cognitive, and environmental status of the individual [7]. Mobile phone sensors have been used to predict depressive symptoms in daily life behavior [8]. Similarly, mobile sensor data has been used to develop the algorithm for predicting falls in elderly patients [9] and patients with lower limb amputation [10]. It had been found that mobile sensor data had increased the accuracy of cough classification and spirometry event classification [11]. All these pieces of evidence suggest that sensor data has the potential to transform personal and institutional care with high-fidelity contextual information [5].

Amongst the various sensor data, acceleration data can determine the activity intensity, body position, and activity type for establishing the relationship with health, which can be used further in clinical practice for decision-making [12]. The accelerometer data have also been used effectively for evaluating the transfer skills of wheelchair users [13].

If clinicians can remotely see the progress of their patients in real-time, it would help them intervene when it is required most and would also help them in creating a robust treatment plan specifically according to the requirements of an individual. This will also reduce the transportation cost for the patient and help them recover faster as they are being monitored continuously by their healthcare providers.

Overall aim of the study

The study aimed to develop and test the intervention protocol for remote monitoring and improving the sedentary lifestyle using mobile sensor data.

Research questions

The study will address the following research questions: 1. Can mobile sensor metadata statistics be used for addressing population health issues related to physical activity? 2. How can a mobile device be used for remote monitoring of patients with sedentary lifestyles? 3. Can a mobile device be used for improving the physical activity of the patient and for clinical decision-making?

Study objectives

The objective of this study is to study and encourage the use of the accelerometer sensor metadata of the mobile device in addressing the challenges related to health care service and facilitating the delivery of interventions related to physical activity. The proposed study will help us to examine the effect of continuous monitoring of accelerometer sensor data in improving physical activity and aiding clinical decision-making.

## Materials and methods

Study design

A sequential explanatory mixed-method research design would be used for collecting the data about the activity level of the subjects and to be monitored by the clinicians for intervening to increase the activity level. This study will be conducted over a period of 15 months.

According to the World Health Organization (WHO), individuals who engage in less than 2.5 hours of moderate physical activity or 1.25 hours of vigorous activity in a week are considered sedentary. Individuals with a sedentary lifestyle between the age group of 30 and 50 and visiting the clinics of enrolled clinicians in Jodhpur for the treatment of lifestyle-related diseases like high blood pressure and type 2 diabetes, participants will be recruited for participation in the study after receiving their consent. Participants with a smartphone would be the obvious choice, as the study demands it. 

A mobile health application will be developed using the open-source digital data collection platform for collecting sensor data from mobile devices to monitor the physical activity of the patient. An interactive dashboard will be created for clinicians to monitor the physical activities of each patient enrolled under them. The dashboard will also alert the clinicians about the low level of physical activity after enrollment and assign them the task of encouraging the patients with the help of various messaging platforms. At the end of the study, the feasibility and utility of the intervention will be evaluated by comparing and analyzing the difference in physical activity data and conducting in-depth discussions with the clinicians and participants.

Study settings 

The study will enroll the subjects with a confirmed diagnosis of type 2 diabetes and hypertension and seek treatment at various hospitals in Jodhpur and Chennai. Screening of the eligible participants will be done using the sedentary lifestyle tool [14, 15], and those meeting the eligibility criteria will be invited to participate in the study.

Subject eligibility 

Subjects between the age groups of 30 and 50 with a sedentary lifestyle visiting physicians for the treatment of lifestyle-related disease will be eligible to PR in the study. As per the study protocol, since it requires collecting mobile accelerometer sensor data, it would be necessary for the participants to have a smartphone. Participants who will sign the informed consent for participating in the study will only be eligible further to get enrolled in the study. The study will exclude the participants who have some other comorbid conditions and who are already enrolled in physical activity improvement tasks like gymming, exercising, and not having a smartphone.

Proposed intervention 

With the help of open-source digital data collection software, a survey questionnaire tool will be developed to monitor the daily physical activity level of the subjects by recording the mobile accelerometer data during a particular time period. The participants will be able to remotely submit the quantitative data with the open-source application after completing the physical activity without visiting the clinics. If needed, participants will also be sensitized to using mobile applications while enrolling them in the study. A dashboard will be created to help the clinician monitor these data and compare them with the advice given by them. The participants will also receive advice and suggestions from the clinician based on their physical activity at certain intervals. This study will further explain the benefit of remote monitoring and its impact on changing the physical activity level with the help of a focused group interview of participants and clinicians. Overall, this study will try to create and validate a prototype for capturing and using the accelerometer sensor data to record the change in overall activity level, increasing the ease of monitoring, and improving clinical decision-making, which can be tested further for other sensor data as well.

Procedures for implementing intervention

Participants will need to submit their physical activity data as suggested by the clinicians. The study proposes to collect data related to physical activity like walking and running, with the help of a smart mobile device.

The clinicians will remotely monitor the sensor data collected about the physical activity of the subjects. The collected data will be viewed through the use of an online interactive internet-enabled dashboard to help the clinician with easy monitoring data and informed decision-making. The clinicians will be able to send reminders and suggestions to the subjects based on their average activity levels.

Informed consent 

After screening the eligibility of participants, a pre-approved informed consent form from the Institutional Review Board (IRB) will be delivered digitally to the subjects to allow them to participate voluntarily in the study and be permitted to use their primary data for research and analysis purposes. The study has already been approved by the University Research Ethics Committee of DIT University, Dehradun, India (approval number: DITU/UREC/2021/07/7). No monetary compensation will be provided to participants for participating in the study, and this will be mentioned clearly in the informed consent form.

Data entry and quality assurance 

In addition to the sensor data, all other data will also be collected digitally; hence, data entry will happen directly to the mobile device and will be synched to the server for further analysis and passing on the information to the clinicians. Data validation measures will be followed to maintain the data quality through the study periods. Proper backups of data and documents will be taken at regular intervals during the research project. Data anonymization protocols will be followed thoroughly to maintain confidentiality. 

Data collection instruments 

Quantitative Data Collection

Sociodemographic data like age, gender, education, employment, and health data for the presence of lifestyle-related diseases will be collected. A pre-validated and reliable sedentary lifestyle score-measuring questionnaire will be used to identify the sedentary lifestyle behavior in subjects (Appendix A). During the intervention phase, physical activity-related sensor data, mainly accelerometer data, will be collected using open-source digital data collection platforms like SurveyCTO. The accelerometer sensor data can measure the directional movement of the mobile device along the linear axis to provide an idea about the physical activity of the subjects carrying that device. 

Qualitative Data Collection

We will conduct in-depth interviews, where clinicians will be required to answer open-ended, one-on-one interviews lasting approximately five to 10 minutes, allowing for a detailed exploration of their experiences, opinions, and behaviors related to the clinicians. This will help in improving the prototype further for designing such kinds of remotely monitored interventions. 

Potential confounders

Data will be recorded on health behaviors, disease status, sedentary lifestyle, and physical activity of the subject. However, they each are dependent on several other confounding factors, like the nature of work, heredity, peer pressure, etc. An improvement in any of the parameters might not be directly related to the intervention proposed in the study.

Outcomes

The proposed study will enhance adherence to the prescribed physical activity regime by clinicians, resulting in an improvement of the physical activity levels of the study participants over a period of a few days. Data will be recorded at baseline and follow-up months one, two, and three. 

Analysis plan 

An interactive dashboard will be created on Google Sheets (Google Inc., Mountain View, CA) for monitoring patient activities. Descriptive analysis will be performed to report the mean and standard deviation for the continuous variable and frequency distribution of the categorical variable. The association between the sensor data and improvement in particular health behavior activities will be conducted using correlation analysis. Additionally, chi-square tests for categorical variables and t-tests or Mann-Whitney U tests for continuous variables, depending on the distribution of the data, will be applied. Content analysis of the qualitative data will be conducted to generate themes that are essential to influencing the physical activity level among patients with confirmed diabetes and hypertension.

Ethics and dissemination

This study will be conducted in adherence to the Ethical Principles for Medical Research Involving Human Subjects, as per the Declaration of Helsinki. The study proposal got approval from the University Research Ethics Committee (UREC) of DIT University, Dehradun, India, with the reference number DITU/UREC/2021/07/7. The findings of the research will be disseminated via poster presentation at national and international conferences and also publishing the articles in peer-reviewed journals. The study will help us assess the usefulness of mobile sensor data in improving clinical decision-making and designing remotely monitored interventions for better clinical outcomes by improving the parameters involved in lifestyle-related diseases. The findings related to the various objectives of the study will be shared from time to time with the university, community, and other stakeholders. Prior permission from the participants will be taken to use the data for various publications. Participants’ identifier data will not be shared outside the study and study staff. The clinicians will be made to sign the data confidentiality agreement before participating in the study.

Research timeline

The research timeline outline shown in Table 1, enumerates the key phases and milestones of the study, ensuring a structured and systematic approach to achieving the project’s objectives. It provides a clear schedule for each stage, including literature review, data collection, analysis, and report writing. By defining specific deadlines for each task, the timeline helps to maintain focus, allocate resources efficiently, and monitor progress. This framework ensures that the research is completed within the allocated time while meeting all critical goals and deliverables.

**Table 1:** Research timeline and milestones

Task	Months			
	1-3	4-6	7-12	12-15
Review of the literature, initial designing, and planning of the study	Yes			
Development of study proposal and ethical approval	Yes			
Development of survey items and the questionnaire	Yes			
Approval of the study proposal		Yes		
Pilot testing of the representative sample of the target population		Yes		
Initial data analysis, results write up, and dissemination of the pilot survey		Yes		
Revision of the questionnaire based on the pilot testing		Yes		
Development of electronic survey Recruitment of the target sample		Yes		
Reviewing collected data by the research team			Yes	
Data analysis			Yes	
Results write-up and preparation of the manuscript				Yes
Dissemination				Yes

## Results

The proposed research will help in exploring the role of remote monitoring of sensor data in improving adherence to physical activity plans as prescribed by their clinicians. It will also help in studying the variables that influence the monitoring of remote data collection.

## Discussion

Despite the free availability of mobile sensor data, its uses are very limited in remote monitoring of activities and for checking the sedentary behavior of an individual. The pace at which these kinds of digital interventions should be introduced is not optimal [16]. Additionally, there is a dearth of existing literature for using sensor data as a monitoring tool, specifically in the context of Indian settings. The proposed study would help in an in-depth understanding of sensor data monitoring and its implication for improving physical activity data monitoring. The study will also provide an understanding of the challenges and barriers associated with sensor data monitoring and using it for public health interventions. Some findings suggest that smartphone-based monitoring of the patient may also help in reducing the workload of clinicians [17]. The outcome of this study will also encourage clinicians to use other sensor data for their day-to-day clinical decision-making activities and, in turn, will also encourage further research on using the sensor data for various public health interventions.

With the introduction of artificial intelligence (AI), the sensor data related to the different activities of an individual can be used to monitor the overall health behavior of the person and predict lifestyle-related diseases to the same. Various AI models can be created for identifying the risks related to lifestyle-related diseases and not only recommending the changes needed to that lifestyle but also tracking the improvements as per the recommendation. It can also be used for clinical trials to measure the safety and efficacy of new drugs, as it can provide a lot of information related to changes in physical activities after and before consuming drugs.

Limitations

However, one of the major limitations of the study is that it will be conducted using mobile sensor data, which is not as accurate as the other sensor-collecting gadgets like smartwatches, wristbands, global positioning system (GPS) tracking devices, and accelerometers, however, it will still give an idea about the basic activity level of the patient and can effectively monitor the changes in the activity level.

Additionally, this study will be conducted in a setting that is part of a developing country; the generalization of the outcomes to the settings of developed countries will not be as straightforward due to factors like education level and easy access to technology. The sample size of the study also may not be representative of the population. Additionally, it will not be possible to measure the effectiveness of the protocol given the non-availability of the existing literature testing similar protocols or to compare the standards of various sensor data collected from the different devices. Moreover, this study will not establish any standardization of the mobile sensor data, which could be very critical for the future of interventions using the sensor data.

## Conclusions

The use of sensor data in public health has a lot of potential to improve behavior monitoring, improving the behavior also, and subsequently improving public health parameters related to health behavior. It can add a new dimension to personalized care through remote monitoring. However, there is a considerable shortage of literature and research to set up the sensor monitoring workflow and establish its advantages. This study can fill the existing void in the literature world about using sensor data for remote monitoring of physical activity and may pave the way for many such studies in the future. This study can also help in creating awareness and encouraging research about the standardization of sensor data, which is the biggest challenge in implementing sensor data monitoring-related interventions. Despite some teething challenges related to data security and privacy, the future of patient monitoring with the collection of sensor data in public health seems very encouraging and promising.

## Figures and Tables

**Figure 1:**
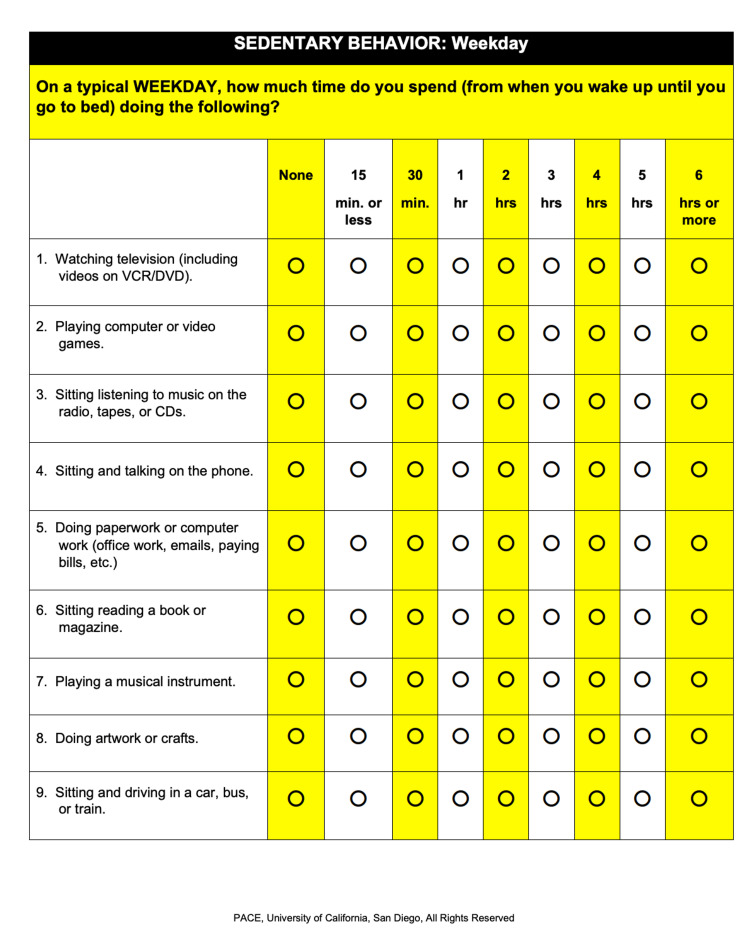
Page 1 of the sedentary lifestyle questionnaire Used with approval from Rosenberg et al. who are the creators of the questionnaire [16].

**Figure 2:**
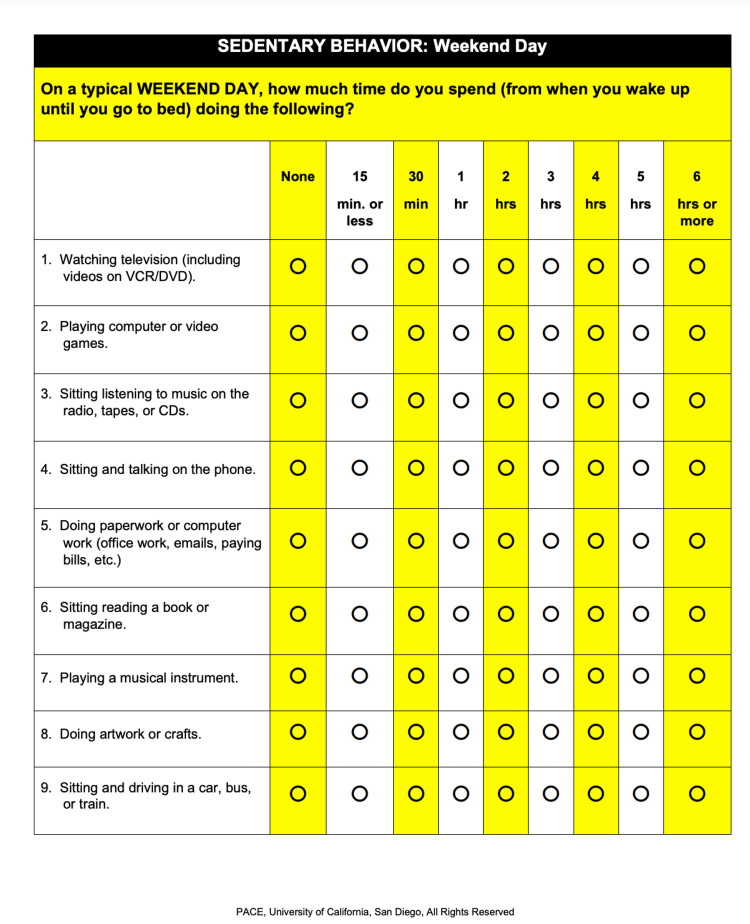
Page 2 of the sedentary lifestyle questionnaire Used with approval from Rosenberg et al. who are the creators of the questionnaire [16].

## Appendices

Appendix A 

The Sedentary Lifestyle Questionnaire
